# The Association Between Single-Nucleotide Polymorphisms of Co-Stimulatory Genes Within Non-HLA Region and the Prognosis of Leukemia Patients With Hematopoietic Stem Cell Transplantation

**DOI:** 10.3389/fimmu.2021.730507

**Published:** 2021-10-04

**Authors:** Ding-Ping Chen, Su-Wei Chang, Po-Nan Wang, Wei-Tzu Lin, Fang-Ping Hsu, Wei-Ting Wang, Ching-Ping Tseng

**Affiliations:** ^1^ Department of Laboratory Medicine, Linkou Chang Gung Memorial Hospital, Taoyuan, Taiwan; ^2^ Department of Medical Biotechnology and Laboratory Science, College of Medicine, Chang Gung University, Taoyuan, Taiwan; ^3^ Graduate Institute of Biomedical Sciences, College of Medicine, Chang Gung University, Taoyuan, Taiwan; ^4^ Clinical Informatics and Medical Statistics Research Center, Chang Gung University, Taoyuan, Taiwan; ^5^ Division of Hematology-Oncology, Department of Internal Medicine, Linkou Chang Gung Memorial Hospital, Taoyuan, Taiwan

**Keywords:** hematopoietic stem cell transplantation, single-nucleotide polymorphism, non-HLA, CTLA4, CD28, TNFSF4, PDCD1 (PD-1)

## Abstract

To avoid graft rejection, the hematopoietic stem cells with matched classical human leukocyte antigen (HLA) alleles are the primary choice for clinical allogeneic transplantation. However, even if the fully HLA-matched hematopoietic stem cells are used for transplantation, some patients still have poor prognosis after hematopoietic stem cell transplantation (HSCT), suggesting that the HLA system was not the only determinant of the outcomes of HSCT. In this study, we investigated whether the single-nucleotide polymorphisms (SNPs) of the co-stimulatory genes within non-HLA regions were related to the outcomes of HSCT. The genomic DNAs of 163 patients who had acute leukemia and received HSCT and their respective donors were collected for analysis. Thirty-four SNPs located in the four co-stimulatory genes including cytotoxic T-lymphocyte associated protein 4 (CTLA4), CD28, tumor necrosis factor ligand superfamily 4 (TNFSF4), and programmed cell death protein 1 (PDCD1) were selected to explore their relationship with the adverse outcomes after transplantation, including mortality, cytomegalovirus infection, graft-versus-host disease, and relapse. Our results revealed that nine SNPs in the CTLA4 gene, five SNPs in the PDCD1 gene, two SNPs in the TNFSF4 gene, and four SNPs in the CD28 gene were significantly associated with the occurrence of adverse outcomes post-HSCT. These SNPs may play important roles in immune response to allografts post-HSCT and can be the targets for developing strategy to identify appropriate donors.

## Introduction

Leukemia is a type of cancer with abnormal blood cells. It can be classified into myeloid and lymphoid lineage depending on the type of aberrantly multiplying cells. It can also be distinguished as acute or chronic according to the rate of disease progression. Acute leukemia has the characteristics of poor survival rate. Nowadays, the main treatment of acute leukemia is hematopoietic stem cell transplantation (HSCT), enabling reconstruction of the immune and hematopoietic systems by transplanting autologous or allogeneic hematopoietic stem cells into patients (1). Human leukocyte antigen (HLA) genes are located on the short arm of human chromosome 6, playing vital roles in immune response to allografts (2, 3). Hence, it is mandatory to confirm that the HLA alleles are matched between recipients and donors before transplantation (3–5), especially the classical HLA genes, such as HLA-A, -B, -C, and -D (-DR, -DQ, -DP). Because these genes are closely linked to each other, the HLA genes are inherited in the form of haplotype (6).

According to the relationship between the donor and recipient, HSCT can be divided into related and unrelated transplantation. In general, the survival rate of the former was higher than that of the latter. The incidence of adverse outcomes, such as acute graft-versus-host disease (GVHD), for related transplantation was generally lower than that of the unrelated transplantation (7, 8). GVHD is one of the complications of allogeneic HSCT caused by the donor’s T cells attacking organs and tissues of recipients (9). Cytomegalovirus (CMV) infection is also a complication of allogeneic HSCT, resulting from the destroyed immune system in patients receiving high-dose treatment regimens (10). Patients with CMV infection and GVHD usually had a higher risk for recurrence and death (11). The allografts obtained from HLA-matched siblings was better than that obtained from the HLA-haploidentical parents, siblings, or unrelated sources (7, 12, 13). Because the probability of having HLA-matched siblings is 25%, there is only about a one-third chance to obtain an HLA-matched related donor. Most patients can only rely on public donation. Nevertheless, the source of donation is limited and patients usually are not able to acquire a suitable donor in time. Because graft failure still occurs even when an HLA-matched sibling was chosen as the donor, additional factors beyond HLA are likely to be involved in the regulator of allograft rejection (14, 15).

The association between HSCT and non-HLA genes such as tumor necrosis factor ligand superfamily 4 (TNFSF4), cytotoxic T-lymphocyte associated protein 4 (CTLA4 or CD152), programmed cell death protein 1 (PDCD1, PD-1 or CD279), and CD28 has been shown in previous studies (16–19). These genes belong to the co-stimulatory system. The imbalance of co-stimulatory molecules is one of the immune escape mechanisms in hematological cancers. It may promote the development of various autoimmune diseases and cancers (20). Several studies indicate that CD28, CTLA4, TNFSF4, and PDCD1 play important roles in the immune system and transplantations (21–23). In addition, genetic variants such as single-nucleotide polymorphisms (SNPs) in HLA and non-HLA regions have been linked to the success or failure of HSCT among different ethnic populations (24, 25). In this study, we explored the association between donor SNPs of co-stimulatory genes (CTLA4, CD28, TNFSF4, and PDCD1) and the mortality, CMV infection, GVHD, and relapse of their corresponding recipients in the Taiwanese population. This study provides new insights into understanding the roles of co-stimulatory genes in prognosis after transplantation and may lead to developing strategy to identify appropriate donors for HSCT.

## Methods and Materials

### Patients and HLA Typing

This study was reviewed and approved by the Institutional Review Board of Chang Gung Memorial Hospital, and its approval IDs were 201304949B0, 201700769B0, 201701849B0, 201801985B0, and 201901246B0. All donors and recipients except the donors of unrelated HSCT signed informed consents. All methods of the study were performed according to the ethical requirements and regulations. For unrelated donors, informed consents were exempted because the HLA-matched donors were selected by the Stem Cells Center in Taiwan and the identity of the donors were made anonymous and disconnected to the physicians and research team. A total of 163 patients receiving HSCT was enrolled in this study, in which 99 patients were diagnosed as acute myeloid leukemia (AML), and 64 patients were acute lymphoblastic leukemia (ALL). The clinical characteristics of the 163 patients are shown in **Table 1**. All donor–recipient pairs had fully matched HLA as revealed by high-resolution HLA typing using the SeCore kit (Thermo Fisher, Waltham, MA). The MicroSSP Allele Specific Typing Tray (Thermo Fisher, Waltham, MA) was used to resolve ambiguous alleles of the SeCore typing with sequence-specific primers.

**Table 1:** Clinical characteristics of patients who enrolled in the study.

Clinical features	No. of patients (%)	No. of AML patients (%)	No. of ALL patients (%)
No. of patients	163	99	64
Median age (years, range)	28 (0.7–66)	33 (1–66)	21 (0.7–54)
Gender (male/female)	75 (46)/88 (54)	42 (42)/57 (58)	33 (52)/31(48)
Mortality	83 (51)	51 (52)	32 (50)
CMV infection	88 (54)	62 (63)	26 (41)
Relapse	71 (44)	47 (47)	24 (38)
Acute GVHD I–II	48 (29)	25 (25)	23 (36)
Acute GVHD III–IV	11 (7)	7 (7)	4 (6)
Chronic GVHD	90 (55)	58 (59)	32 (50)
No GVHD	14 (9)	9 (9)	5 (8)

AML, acute myeloid leukemia; ALL, acute lymphoblastic leukemia; CMV, cytomegalovirus; GVHD, graft-versus-host disease.

### Definition of Outcomes

Mortality was referred to the state of patients who died in the duration of study. The presence of CMV antigen or DNA in the peripheral blood of recipients after transplantation was defined as a CMV-infected case. CMV antigen in the leukocytes was determined by CMV Antigenemia Assay (MONOFLUO™, Bio-Rad). The test was considered positive when more than two polymorphonuclear leukocytes (PMN) were positive for CMV antigen in a total of 50,000 PMN. CMV DNA Quantitative Amplification test is a real-time quantitative PCR assay (COBAS^®^ AmpliPrep/COBAS^®^ TaqMan^®^ CMV Test, Roche). The nucleic acid test was considered positive when the Ct < 37. These two assays can assist clinicians in monitoring the status of CMV infection.

According to the International Bone Marrow Transplant Registry, GVHD was considered as acute GVHD (aGVHD) when it occurred within 100 days after transplantation. It can be divided into four grades according to the clinical characteristics of organs as defined below. Grade I: maculopapular rash over <25% of body area with no liver or gastrointestinal involvement; Grade II: maculopapular rash over 25% to 50% of body area, diarrhea 500 to 1500 ml/day, and bilirubin 2 to 6 mg/dl; Grade III: maculopapular rash over >50% of body area, and severe diarrhea; Grade IV: skin blisters, bilirubin >15 mg/dl, severe diarrhea with pain, and life-threatening. Grades I–II were defined as mild GVHD, and Grades III–IV were defined as severe GVHD. Chronic GVHD (cGVHD) usually occurs more than 100 days after transplantation or occurs continually for more than 100 days without remission (26). Patients without any symptoms of aGVHD or cGVHD during the study period were defined as no GVHD.

Relapse was defined as recurrence of malignancy based on one or more of the following: bone marrow morphology, minimal residual disease by either flow cytometry, cytogenetics, imaging results, or short tandem repeat (STR) analysis. High-throughput amplicon sequencing (AmpFISTR Identifiler Amplification Kit, Thermo Fisher, Waltham, MA) was performed to analyze STR and to evaluate HSCT engraftments for identification of mixed chimerism (27–29) according to the manufacturer’s instruction. The presence of >5% recipient STR alleles in the chimeric test was considered as a surrogate marker of disease relapse.

### Selection of SNPs

Based on our initial screening of SNPs that were present in the CTLA4 gene of the Taiwanese population, and the studies demonstrating the importance of promoter and exon 1 in gene expression and the SNPs with clinical association (30), a total of eight DNA fragments of the four co-stimulatory genes (CTLA4, TNFSF4, CD28, and PDCD1) were selected for analyses of donor SNPs (**Table 2**). A total of 17 SNPs in CTLA4, 3 SNPs in TNFSF4, 9 SNPs in CD28, and 5 SNPs in PDCD1 were subject to association study with the risk for relapse, mortality, GVHD, and CMV infection. All SNP variants were deposited to the NCBI database dbSNP and the accession numbers are provided in the **Supplementary Table S1**. Data can be accessed with the following link: https://www.ncbi.nlm.nih.gov/SNP/snp_viewTable.cgi?handle=WANGWT.

**Table 2:** The 34 SNPs for association study with post-HSCT adverse outcomes.

Gene	Genomic region	SNP under analysis			
CTLA4	Promoter	rs11571315	rs733618	rs4553808	rs11571316
		rs62182595	rs573554201	rs16840252	rs945677329
		rs5742909			
					
	Exon 1	rs231775			
					
	Exon 4	rs56102377	rs56217811	rs55696217	
					
	3’-UTR	rs231721	rs778932058	rs3087243	rs11571319
					
TNFSF4	Promoter	rs1234314	rs45454293	rs181758110	
					
CD28	Promoter	rs1879877	rs3181096	rs3181097	rs3181098
		rs28718975	rs28688913	rs28541784	rs20189072
		rs200353921			
					
PDCD1	Promoter	rs5839828	rs36084323		
					
	Intron 4	rs41386349	rs6705653		
					
	Exon 5	rs2227982			

### PCR and Sequencing

Peripheral blood (3 ml) was collected from the corresponding donors of the recipients, and the genomic DNA was extracted by using QIAamp DNA Blood Mini Kit (Qiagen, Valencia, California, USA). A total of eight primer pairs (**Table 3**) were used to amplify genomic DNA fragments covering the 34 SNPs as abovementioned (**Table 2**). The PCR reaction volume was 25 μl, including 1 μl of forward and reverse primer (10 μM), 12.5 μl of 2X HotStart PCR Mix (BIOMAN, Taipei, Taiwan) containing HotStart Taq DNA polymerase, reaction buffer, and dNTP, 1 μl of sample DNA and 9.5 μl of ddH_2_O. The PCR program was 1 cycle at 94°C for 4 min, 30 cycles of 94°C for 30 s, 58°C for 30 s, 72°C for 45 s, and 1 cycle of 72°C for 10 min. Subsequently, 5 μl of PCR products was fractionated on a 1.5% or 2% agarose gel to visualize the PCR products. The size of PCR products ranged from 1,039 bp to 2,234 bp (**Table 3**). The Big Dye Terminator Cycle Sequencing kit (Thermo Fisher, Waltham, Massachusetts, USA) and the ABI PRISM genetic analyzer (Thermo Fisher, Waltham, Massachusetts, USA) were used for direct sequencing according to the manufacturer’s instructions. Because of the insufficient genomic DNA and failure of PCR reaction, not every donor had complete SNP data available.

**Table 3:** Primer sequences for amplification of candidate SNPs.

Gene name	Genomic region	Primer sequence	PCR product (bp)
CTLA4	Promoter	F: 5’-GGCAACAGAGACCCCACCGTT-3’	1233
		R: 5’-GAGGACCTTCCTTAAATCTGGAGAG-3’	
	Promoter	F: 5’-CTCTCCAGATTTAAGGAAGGTCCTC-3’	1169
	and exon 1	R: 5’-GGAATACAGAGCCAGCCAAGCC-3’	
	Exon 4	F: 5’- CTAGGGACCCAATATGTGTTG-3’	1039
		R: 5’-AGAAACATCCCAGCTCTGTC-3’	
	Exon 4	F: 5’-GCTTGGAAACTGGATGAGGTCATAGC-3’	1204
	and 3’-UTR	R: 5’-AGAGGAAGAGACACAGACAGAGTTGC-3’	
TNFSF4	Promoter	F: 5’-GGCTTGGAGTCTATGATATTGTGCC-3’	1725
	and exon 1	R: 5’-GAAGGGCGTTTAACCACACTTTACG-3’	
CD28	Promoter	F: 5’- GGGTGGTAAGAATGTGGATGAATC-3’	1961
	and exon 1	R: 5’-CAAGGCATCCTGACTGCAGCA-3’	
PDCD1	Promoter	F: 5’-ACCCACACAGCCTCACATCTCT-3’	1778
	and exon 1	R: 5’-AAACTGAGGGTGGAAGGTCCCT-3’	
	Exon 4, intron 4	F: 5’-TGGTGACCCCAAGTGTGTTTCTC-3’	2234
	and exon 5	R: 5’-GAGGAATTTTTCACCGGAGGGC-3’	

F, forward primer; R, reversed primer.

### Statistical Analysis

Single-locus association tests were performed to identify the donor SNPs that were associated with the defined outcomes in the recipients. The allele or genotype frequencies between cases (patients with the indicated outcomes) and controls (patients without the indicated outcomes) were compared with Cochran-Armitage Trend test (or Trend test) and the allelic test using the PLINK software v1.07 (31). The allele effects of the SNPs on each outcome were further examined using the logistic regression analysis assuming three modes of inheritance: additive model, recessive model, or dominant models. *p* < 0.05 was considered statistically significant. The Haploview 4.2 (32) software was used to determine the linkage disequilibrium (LD). The pairwise linkage disequilibrium value D’ and the haplotype blocks of SNPs were determined. The haplotype blocks were defined as the SNPs in this region had no evidence for historical recombination.

## Results

### Patient Characteristics and Study Design

A total of 163 patients receiving HSCT including 99 patients with AML and 64 patients with ALL were enrolled in this study. Clinical characteristics and the tracking data of mortality, CMV infection, relapse, and GVHD for these patients are listed in **Table 1**.

With the importance of co-stimulatory signals in the immune system and transplantation tolerance, the associations of the four co-stimulatory genes including CTLA4, TNFSF4, CD28, and PDCD1 with the mortality, relapse, CMV infection, and GVHD after HSCT were analyzed in this study. Based on our initial screening of SNPs in the CTLA4 gene of the Taiwanese population, the importance of promoter and exon 1 in gene expression, and the SNPs with clinical association, the genomic regions covering the promoter, exon 1, exon 4, and 3’-UTR of CTLA-4 (17 SNPs), the promoter and exon 1 of TNFSF4 (3 SNPs), the promoter and exon 1 of CD28 (9 SNPs), and the promoter, exon 1, and exons 4–5 including intron 4 of PDCD1 (5 SNPs) for all donors were amplified by PCR using the forward and reverse primers (**Tables 2**, **3**). The PCR amplicons were sequenced and the association of candidate SNPs with the adverse outcomes of patients with AML and ALL were analyzed by Trend test and allelic test, and by logistic regression analysis with additive, dominant, or recessive mode, respectively. The genotype and allele frequencies for all donors are summarized in the **Supplementary Tables S2** **–** **S5**.

### Association of Donor SNPs With the Mortality, CMV Infection, and Relapse of Patients With AML and ALL

By analyzing a total of 34 SNPs, 4 SNPs (rs733618, rs11571316, and rs3087243 in CTLA-4, and rs41386349 in PDCD1) and 1 SNP (rs11571315) elicited significant recessive allelic effects and contributed to the post-HSCT mortality for patients with AML and ALL, respectively (**Table 4**). For patients with AML, the C allele of rs733618 (CC vs. CT+TT, *p* = 0.0376, OR = 2.77, and 95% CI = 1.07–7.22) and the G allele of rs11571316 (GG vs. AA+AG, *p* = 0.0441, OR = 2.32, and 95% CI = 1.03–5.24) located in the promoter region of CTLA4 were associated with higher risk for mortality. The SNP rs3087243 in the 3’-UTR of CTLA4 (GG vs. AA+AG, *p* = 0.0441, OR = 2.32, and 95% CI = 1.03–5.24) and rs41386349 in the intron 4 of PDCD1 (GG vs. AA+AG, *p* = 0.0362, OR = 2.62, and 95% CI = 1.07–6.42) also conferred recessive effects to the risk for mortality of patients with AML. In addition, the SNP of rs41386349 also elicited additive effects on post-HSCT mortality for patients with AML. For patients with ALL, the C allele of rs11571315 (CC vs. CT+TT, *p* = 0.0289, OR = 6.14, 95% CI = 1.21–30.99) in the promoter region of CTLA4 was found to associate with higher risk for mortality.

**Table 4:** The SNPs associated with the mortality post-HSCT.

SNP	Gene position	Risk allele no. (%)	No. of patients (%)			Trend *p*	Allele *p*	OR	Model	Logistic regression *p*	OR
**AML**											
rs733618	CTLA4 (-1722) promoter	C	C/C	C/T	T/T	0.1066	0.0757	1.69 (0.95-3.02)	Additive	0.1072	1.56 (0.91–2.66)
Case		52 (58.43)	18 (69.23)	16 (43.24)	14 (46.67)				Dominant	0.5106	1.34 (0.56–3.19)
Control		37 (41.57)	8 (30.77)	21 (56.76)	16 (53.33)				Recessive	0.0376	2.77 (1.07–7.22)
rs11571316	CTLA4 (-1577) promoter	G	A/A	A/G	G/G	0.0652	0.0706	1.82 (0.95-3.47)	Additive	0.0596	1.96 (0.98–3.95)
Case		79 (56.03)	2 (40)	17 (41.46)	31 (62)				Dominant	0.5821	1.67 (0.27–10.4)
Control		62 (43.97)	3 (60)	24 (58.54)	19 (38)				Recessive	0.0441	2.32 (1.03–5.24)
rs3087243	CTLA4 (ct60) 3’-UTR	G	A/A	A/G	G/G	0.0621	0.0706	1.82 (0.95-3.47)	Additive	0.0596	1.96 (0.98–3.95)
Case		79 (56.03)	2 (40)	17 (41.46)	31 (62)				Dominant	0.5821	1.67 (0.27–10.4)
Control		62 (43.97)	3 (60)	24 (58.54)	19 (38)				Recessive	0.0441	2.32 (1.03–5.24)
rs41386349	PDCD1 (IVS4+251) intron 4	G	A/A	A/G	G/G	0.0289	0.0201	2.45 (1.16-5.2)	Additive	0.0267	2.37 (1.11–5.08)
Case		77 (57.04)	1 (20)	11 (40.74)	33 (61.11)				Dominant	0.1713	4.76 (0.52–43.93)
Control		58 (42.96)	4 (80)	16 (59.26)	21 (38.89)				Recessive	0.0362	2.62 (1.07–6.42)
**ALL**											
rs11571315	CTLA4 (-1765) promoter	C	C/C	C/T	T/T	0.0833	0.0498	2.08 (1-4.31)	Additive	0.0678	1.95 (0.96–3.97)
Case		29 (60.42)	9 (81.82)	11 (42.31)	11 (42.31)				Dominant	0.3598	1.6 (0.59–4.39)
Control		19 (39.58)	2 (18.18)	15 (57.69)	15 (57.69)				Recessive	0.0289	6.14 (1.21–30.99)

One SNP (rs6705653 in PDCD1) and four SNPs (rs36084323, rs41386349, rs6705653, and rs2227982 in PDCD1) were found to associate with CMV infection in patients with AML and ALL, respectively (**Table 5**). For patients with AML, the C allele of rs6705653 (CC+CT vs. TT, *p* = 0.0138, OR = 7.91, and 95% CI = 1.54–40.71) elicited a dominant effect and contributed to the risk for CMV infection. For patients with ALL, the alternative T allele of the same SNP rs6705653 elicited an additive (Trend test: *p* = 0.0198, and additive effect: *p* = 0.0186) or a dominant effect to the risk for CMV infection (TT+CT vs. CC, *p* = 0.0201, OR = 3.95, and 95% CI = 1.25–12.49). The C allele of rs36084323, A allele of rs41386349, and G allele of rs2227982 in PDCD1 gene also associated with a higher risk for CMV infection (allele model: *p* = 0.0265, 0.0356, and 0.0252, respectively).

**Table 5:** The SNPs associated with CMV infection post-HSCT.

SNP	Gene position	Risk allele no. (%)	No. of patients (%)			Trend *p*	Allele *p*	OR	Model	Logistic regression *p*	OR
**AML**											
rs6705653	PDCD1 (IVS4+541) intron 4	C	C/C	C/T	T/T	0.0947	0.0633	1.92 (0.97–3.82)	Additive	0.0852	1.78 (0.93–3.44)
Case		83 (68.60)	31 (67.39)	21 (72.41)	2 (22.22)				Dominant	0.0138	7.91 (1.54–40.71)
Control		38 (31.40)	15 (32.61)	8 (27.59)	7 (77.78)				Recessive	0.5139	1.35 (0.55–3.29)
**ALL**											
rs36084323	PDCD1 (-606) promoter	C	C/C	C/T	T/T	0.054	0.0265	2.39 (1.11–5.15)	Additive	0.0413	2.2 (1.04–4.69)
Case		28 (51.85)	9 (60)	10 (41.67)	4 (23.53)				Dominant	0.0854	3.09 (0.86–11.08)
Control		26 (48.15)	6 (40)	14 (58.33)	13 (76.47)				Recessive	0.0874	2.89 (0.86–9.72)
rs41386349	PDCD1 (IVS4+251) intron 4	A	A/A	A/G	G/G	0.0609	0.0356	2.86 (1.08–7.56)	Additive	0.0515	2.64 (1–6.99)
Case		13 (61.90)	2 (66.67)	9 (60)	12 (31.58)				Dominant	0.0399	3.4 (1.06–10.89)
Control		8 (38.10)	1 (33.33)	6 (40)	26 (68.42)				Recessive	0.3752	3.05 (0.26–35.33)
rs6705653	PDCD1 (IVS4+541) intron 4	T	C/C	C/T	T/T	0.0198	0.0100	3.04 (1.31–7.04)	Additive	0.0186	2.81 (1.19–6.61)
Case		21 (61.76)	7 (26.92)	11 (55)	5 (71.43)				Dominant	0.0201	3.95 (1.25–12.49)
Control		13 (28.24)	19 (73.08)	9 (45)	2 (28.57)				Recessive	0.1269	3.89 (0.69–22.03)
rs2227982	PDCD1 (+699) exon 5	G	A/A	A/G	G/G	0.0534	0.0252	2.53 (1.13–5.67)	Additive	0.0541	2.06 (0.99–4.27)
Case		30 (57.69)	4 (26.67)	8 (47.06)	11 (61.11)				Dominant	0.0797	3.27 (0.88–12.19)
Control		23 (42.31)	11 (73.33)	9 (52.94)	7 (38.89)				Recessive	0.1120	2.62 (0.8–8.54)

One SNP (rs200353921 in CD28) and three SNPs (rs5839828, rs36084323, and rs2227982 in PDCD1) were associated with the risk of disease relapse in patients with AML and ALL, respectively (**Table 6**). For patients with AML, the T allele of rs200353921 located on the promoter region of CD28 gene was associated with a higher risk of relapse (allele model *p* = 0.0343 for T vs. A, OR = 2.1, and 95% CI = 1.06–4.18). For patients with ALL, the G7 allele of rs5839828, the C allele of rs36084323, and the G allele of rs2227982 in the PDCD1 gene were also associated with a higher risk for disease relapse (allele model: *p* = 0.0008, 0.0095, and 0.0018, respectively).

**Table 6:** The SNPs associated with disease relapse post-HSCT.

SNP	Gene position	Risk allele no. (%)	No. of patients (%)			Trend *p*	Allele *p*	OR	Model	Logistic regression *p*	OR
**AML**											
rs200353921	CD28 (−879) promoter	T	A/A	A/T	T/T	0.1437	0.0343	2.1 (1.06–4.18)	Additive	0.1187	1.5 (0.9–2.5)
Case		58 (48.33)	7 (30.43)	2 (33.33)	28 (49.12)				Dominant	0.1586	2.08 (0.76–5.71)
Control		62 (51.67)	16 (69.57)	4 (66.67)	29 (50.88)				Recessive	0.1125	2.15 (0.84–5.48)
**ALL**											
rs5839828	PDCD1 (−763) promoter	G7	G6	G6/G7	G7	0.0011	0.0008	3.8 (1.75–8.48)	Additive	0.0024	4.02 (1.64–9.83)
Case		27 (56.25)	3 (14.29)	11 (42.31)	8 (72.73)				Dominant	0.0088	6.33 (1.6–25.04)
Control		21 (43.75)	18 (85.71)	15 (57.69)	3 (27.27)				Recessive	0.0140	6.29 (1.46–27.05)
rs36084323	PDCD1 (−606) promoter	C	C/C	C/T	T/T	0.0206	0.0095	2.8 (1.29–6.17)	Additive	0.0179	2.6 (1.18–5.7)
Case		28 (51.85)	9 (60)	10 (41.67)	3 (17.65)				Dominant	0.0366	4.43 (1.11–17.78)
Control		26 (48.15)	6 (40)	14 (58.33)	14 (82.35)				Recessive	0.0606	3.23 (0.96–10.92)
rs2227982	PDCD1 (+699) exon 5	G	A/A	A/G	G/G	0.007	0.0018	3.9 (1.67–9.35)	Additive	0.0083	3.01 (1.33–6.8)
Case		29 (54.72)	3 (20)	5 (29.41)	12 (66.67)				Dominant	0.0682	3.78 (0.91–15.64)
Control		24 (45.28)	12 (80)	12 (70.59)	6 (33.33)				Recessive	0.0055	6 (1.7–21.13)

### Association of Donor SNPs With the Status of GVHD in Patients With AML and ALL

The status of GVHD was classified into four categories including GVHD III–IV (severe GVHD), GVHD I–II (mild GVHD), chronic GVHD (cGVHD), and no GVHD. Two SNPs (rs1234314 and rs45454293) in the promoter region of TNFSF4 were associated with the risk for severe GVHD (**Table 7**) in patients with AML (rs1234314: Trend test, *p* = 0.006; allele model, *p* = 0.0114 for C vs. G, OR = 7.39, and 95% CI = 1.58–34.52; and rs45454293: Trend test, *p* = 0.0145, allele model *p* = 0.0100 for T vs. C, OR = 4.86, and 95% CI = 1.47–16.07). Both SNPs elicited additive and recessive effects on the risk for severe GVHD. No SNP was found to associate with the risk for severe GVHD in patients with ALL.

**Table 7:** The SNPs associated with GVHD post-HSCT.

SNP	Gene position	Risk allele no. (%)	No. of patients (%)			Trend *p*	Allele *p*	OR	Model	Logistic regression *p*	OR
**GVHD III–IV**											
**AML**											
rs1234314	TNFSF4 (−738) promoter	C	C/C	C/G	G/G	0.006	0.0114	7.39 (1.58–34.52)	Additive	0.0148	7.19 (1.48–34.86)
Case		10 (12.99)	4 (22.22)	2 (4.88)	0 (0)				Dominant	0.9475	n/a
Control		67 (81.01)	14 (77.78)	39 (95.12)	30 (100)				Recessive	0.0123	9.86 (1.66–58.62)
rs45454293	TNFSF4 (−582) promoter	T	C/C	C/T	T/T	0.0145	0.0100	4.86 (1.47–16.07)	Additive	0.0162	4.77 (1.34–16.96)
Case		6 (17.65)	2 (3.45)	2 (7.69)	2 (50)				Dominant	0.1039	4.31 (0.75–24.81)
Control		28 (82.35)	56 (96.55)	24 (92.31)	2 (50)				Recessive	0.0077	20 (2.24–178.9)
**GVHD I–II**											
**ALL**											
rs231775	CTLA4 (+49) exon 1	A	A/A	A/G	G/G	0.0719	0.0343	2.28 (1.07–4.89)	Additive	0.0543	2.05 (0.99–4.26)
Case		22 (45.83)	5 (41.66)	12 (50)	4 (16)				Dominant	0.0156	4.7 (1.35–16.35)
Control		26 (54.17)	7 (58.34)	12 (50)	21 (84)				Recessive	0.5572	1.47 (0.41–5.34)
rs41386349	PDCD1 (IVS4+251) intron 4	A	A/A	A/G	G/G	0.0604	0.0436	2.71 (1.03–7.1)	Additive	0.0613	2.51 (0.96–6.53)
Case		12 (57.14)	3 (100)	6 (40)	12 (25)				Dominant	0.1874	2.17 (0.69–6.8)
Control		9 (42.86)	0 (0)	9 (60)	26 (75)				Recessive	0.9754	n/a
rs6705653	PDCD1 (IVS4+541) intron 4	T	C/C	C/T	T/T	0.0086	0.0039	3.53 (1.51–8.28)	Additive	0.0091	3.29 (1.35–8.02)
Case		19 (55.88)	5 (19.23)	9 (45)	5 (71.43)				Dominant	0.0165	4.52 (1.33–15.43)
Control		15 (44.12)	21 (80.77)	11 (55)	2 (28.57)				Recessive	0.0517	5.71 (1–32.79)
rs2227982	PDCD1 (+699) exon 5	G	A/A	A/G	G/G	0.0194	0.0055	3.4 (1.44–8.03)	Additive	0.0178	2.63 (1.19–5.85)
Case		27 (50.94)	2 (13.33)	7 (41.18)	10 (55.56)				Dominant	0.0291	6.14 (1.21–31.06)
Control		26 (49.06)	13 (86.67)	10 (58.82)	8 (44.44)				Recessive	0.0595	3.19 (0.96–10.62)
**Chronic GVHD**											
**ALL**											
rs5742909	CTLA4 (−319) promoter	C	C/C	C/T	T/T	0.0509	0.0465	3.91 (1.03–14.86)	Additive	0.0541	3.82 (0.98–14.87)
Case		61 (53.98)	29 (56.86)	3 (27.27)	0 (0)				Dominant	0.9856	n/a
Control		52 (46.02)	22 (43.14)	8 (72.73)	1 (100)				Recessive	0.0576	3.95 (0.96–16.23)
rs231775	CTLA4 (+49) exon 1	G	A/A	A/G	G/G	0.1692	0.1049	1.84 (0.88–3.82)	Additive	0.1377	1.69 (0.85–3.37)
Case		42 (56.76)	6 (50)	8 (33.33)	17 (68)				Dominant	0.9495	1.04 (0.3–3.66)
Control		32 (43.24)	6 (50)	16 (66.67)	8 (32)				Recessive	0.0279	3.34 (1.15–9.73)
rs28541784	CD28 (−891) promoter	T	C/C	C/T	T/T	0.0473	0.0303	2.78 (1.11–6.98)	Additive	0.0495	2.46 (1.01–6.02)
Case		19 (70.37)	15 (41.67)	11 (64.71)	4 (80)				Dominant	0.0536	3 (0.99–9.1)
Control		8 (29.63)	21 (58.33)	6 (35.29)	1 (20)				Recessive	0.2161	4.15 (0.44–39.21)
rs6705653	PDCD1 (IVS4+541) intron 4	C	C/C	C/T	T/T	0.0114	0.0066	3.36 (1.41–8.02)	Additive	0.0131	3.12 (1.28–7.63)
Case		42 (58.33)	17 (65.38)	8 (40)	1 (14.29)				Dominant	0.0792	7.14 (0.8–63.38)
Control		30 (41.67)	9 (34.62)	12 (60)	6 (85.71)				Recessive	0.0220	3.78 (1.22–11.71)
rs2227982	PDCD1 (+699) exon 5	A	A/A	A/G	G/G	0.0795	0.0305	2.43 (1.09–5.42)	Additive	0.0624	1.99 (0.97–4.1)
Case		28 (59.57)	10 (66.67)	8 (47.06)	6 (33.33)				Dominant	0.1240	2.57 (0.78–8.51)
Control		19 (40.43)	5 (33.33)	9 (52.94)	12 (66.67)				Recessive	0.0897	3 (0.85–10.6)
**No GVHD**											
**AML**											
rs3181096	CD28 (−1328) promoter	T	C/C	C/T	T/T	0.0849	0.0682	2.66 (0.93–7.58)	Additive	0.0844	2.47 (0.89–6.88)
Case		7 (15.56)	1 (1.85)	7 (22.58)	0 (0)				Dominant	0.0231	11.97 (1.42–100.74)
Control		38 (84.44)	53 (98.15)	24 (77.42)	7 (100)				Recessive	0.9722	n/a
rs3181098	CD28 (−1042) promoter	A	A/A	A/G	G/G	0.0889	0.0723	2.63 (0.92–7.5)	Additive	0.0898	2.43 (0.88–6.75)
Case		7 (15.56)	0 (0)	7 (23.33)	1 (1.92)				Dominant	0.0235	11.9 (1.41–100.34)
Control		38 (84.44)	7 (100)	23 (76.67)	51 (98.08)				Recessive	0.9717	n/a
**ALL**											
rs4553808	CTLA4 (−1661) promoter	G	A/A	A/G	G/G	0.0747	0.0452	4.63 (1.04–20.58)	Additive	0.0627	4.42 (0.93–21.01)
Case		3 (23.77)	3 (5.77)	1 (9.09)	1 (100)				Dominant	0.2255	3.27 (0.49–21.94)
Control		10 (76.23)	49 (94.23)	10 (90.91)	0 (0)				Recessive	0.9957	n/a
rs62182595	CTLA4 (−1478) promoter	A	A/A	A/G	G/G	0.0599	0.0330	5.19 (1.15–23.4)	Additive	0.0515	4.77 (1–22.83)
Case		3 (25)	1 (100)	1 (10)	3 (5.66)				Dominant	0.1824	3.7 (0.55–25.14)
Control		9 (75)	0 (0)	9 (90)	50 (94.34)				Recessive	0.9957	n/a
rs16840252	CTLA4 (−1147) promoter	T	C/C	C/T	T/T	0.0678	0.0350	5.1 (1.13–22.98)	Additive	0.0538	4.7 (0.98–22.49)
Case		3 (25)	3 (5.77)	1 (10)	1 (100)				Dominant	0.1894	3.63 (0.53–24.64)
Control		9 (75)	49 (94.23)	9 (90)	0 (0)				Recessive	0.9953	n/a
rs5742909	CTLA4 (−319) promoter	T	C/C	C/T	T/T	0.0722	0.0479	4.54 (1.02–20.2)	Additive	0.0655	4.36 (0.92–20.69)
Case		3 (23.77)	3 (5.88)	1 (9.09)	1 (100)				Dominant	0.2337	3.2 (0.48–21.5)
Control		10 (76.23)	48 (94.12)	10 (90.91)	0 (0)				Recessive	0.9953	n/a

No SNP was associated with the risk for mild GVHD in patients with AML. Four SNPs (rs231775 in CTLA4, and rs41386349, rs6705653, and rs2227982 in PDCD1) were associated with the risk for mild GVHD (GVHD I–II) in patients with ALL. The A allele of rs231775 on exon 1 of CTLA4 (allele model: *p* = 0.0343 for A vs. G, OR = 2.28, and 95% CI = 1.07–4.89), the A allele of rs41386349 (allele model: *p* = 0.0436 for A vs. G, OR = 2.71, and 95% CI = 1.03–7.1), the T allele of rs6705653 (Trend test: *p* = 0.0086; allele model: *p* = 0.0039 for T vs. C, OR = 3.53, and 95% CI = 1.51–8.28) in the intron 4 of PDCD1, and the G allele of rs2227982 (Trend test: *p* = 0.0194; allele model: *p* = 0.0055 for G vs. A, OR = 3.4, and 95% CI = 1.44–8.03) in the exon 5 of PDCD1 gene contributed to a higher risk for mild GVHD. The three SNPs in the PDCD1 gene were also associated with CMV infection as above mentioned.

No SNP was found to associate with the risk for cGVHD in patients with AML. Five SNPs (rs5742909 and rs231775 in CTLA4, rs28541784 in CD28, and rs6705653 and rs2227982 in PDCD1) were associated with the risk for cGVHD in patients with ALL. The C allele of rs5742909 (allele model: *p* = 0.0465 for C vs. T) and the G allele of rs231775 (recessive model: *p* = 0.0279 for GG vs. AA+AG) on the CTLA4 gene contributed to the higher risk for cGVHD, yet in different modes. In addition, the T allele of rs28541784 on CD28 gene was associated with a higher risk for cGVHD (Trend test: *p* = 0.0473; allele model: *p* = 0.0303 for T vs. C, OR = 2.78, and 95% CI = 1.11–6.98). Of the SNPs located in the PDCD1 gene, the C allele of rs6705653 (allele model: *p* = 0.0066 for C vs. T) and the A allele of rs2227982 (allele model: *p* = 0.0305 for A vs. G) also contributed to the higher risk for cGVHD.

Two SNPs (rs3181096 and rs3181098 in CD28) and four SNPs (rs4553808, rs62182595, rs16840252, and rs5742909 in CTLA4) were associated with the protective effects on the development of GVHD in patients with AML and ALL, respectively. The dominant effects of the T allele of rs3181096 and the A allele of rs3181098 in the promoter region of CD28 conferred protective effects on the development of GVHD in patients with AML (dominant model: *p* = 0.0231 and 0.0235, respectively). The G allele of rs4553808 (allele model: *p* = 0.0452 for G vs. A, OR = 4.63, and 95% CI = 1.04–20.58), the A allele of rs62182595 (allele model: *p* = 0.0330 for A vs. G, OR = 5.19, and 95% CI = 1.15–23.4), the T allele of rs16840252 (allele model: *p* = 0.0350 for T vs. C, OR = 5.1, and 95% CI = 1.13–22.98), and the T allele of rs5742909 (allele model: *p* = 0.0479 for T vs. C, OR = 4.54, and 95% CI = 1.02–20.2) of the CTLA4 gene conferred better protective effects on the development of GVHD in patients with ALL.

### Linkage Disequilibrium

**Figure 1:**
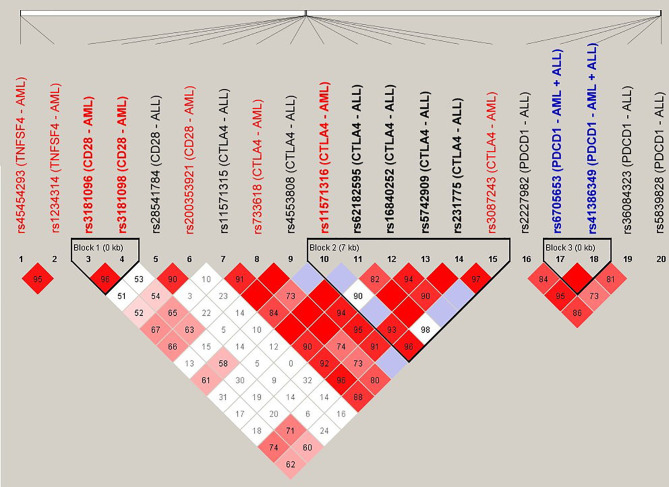
Linkage disequilibrium (LD) analysis of the donor SNPs that were associated with the adverse outcomes of patients with AML and ALL. The pairwise linkage disequilibrium (D’) was given for each pair of SNPs. The red boxes indicated that the pairs of SNPs had high LD, and the lighter the color, the smaller the LD was.

The SNPs (*n* = 20) that were associated with the risk for adverse outcomes in patients with either AML or ALL were subject to LD analysis (**Figure 1**). Several pairs of SNPs had high or complete LD including the rs3087243 with rs231775 (D’ = 0.97), with rs62182595 (D’ = 0.98), and with rs11571316 (D’ = 0.96) in the CTLA4 gene; the rs6705653 with rs41386349 (D’ = 1) in the PDCD1 gene; and the rs3181096 with rs3181098 (D’ = 0.96) in the CD28 gene. In addition, there were three haplotype blocks including the SNPs in the CD28, CTLA4 and PDCD1, respectively. These data imply a potential genetic linkage of these SNPs in the human genome.

## Discussion

The SNPs located in the HLA regions have been reported to associate with the post-HSCT adverse outcomes (33). In this study, we investigated further whether these is an association between 34 donor SNPs in the four co-stimulatory genes (TNFSF4, CTLA4, CD28, and PDCD1) and the occurrence of adverse outcomes (mortality, relapse, CMV infection, and GVHD) for patients with AML and ALL. Our data revealed that 10 and 12 SNPs located in these four genes were related to the adverse outcomes of patients with AML and ALL, respectively.

Co-stimulatory molecules play a critical role in immune regulation and are involved in the pathogenesis of autoimmune diseases, cancers, and graft rejection (34). During T-cell activation, CD28 provides a stimulatory signal when it interacts with CD80/CD86 on the antigen-presenting cells. CTLA4 is then expressed on the activated T cell, playing a role in negative regulation of T-cell activation by competing with CD28 for CD80/CD86 to prevent excessive T-cell activation (35, 36). PDCD1, like CTLA4, plays a negative regulatory role in T-cell activation to develop immune tolerance, which can prevent the development of autoimmune diseases or prevent the immune system from killing cancer cells (37). In addition, the OX40 ligand encoded by TNFSF4 is the key to coordinate innate and adaptive immune cells and plays an important role in the life cycle of immune cells, such as differentiation, activation, inhibition, and apoptosis (38).

Several findings were noted in this study. Most adverse outcomes-related SNPs are unique to patients with AML and ALL, except rs6705653, which is associated with CMV infection for both leukemic types. These data imply that the four co-stimulatory molecules may elicit various functional activity toward AML and ALL cancer cells. In addition, several SNPs are related to more than one clinical outcome in patients with ALL. The SNP of rs41386349 is related to the risk for CMV infection and GVHD I–II, rs36084323 is related to CMV infection and relapse, rs6705653 is related to CMV infection, GVHD I–II, and chronic GVHD, and rs2227982 is related to CMV infection, relapse, GVHD I–II, and chronic GVHD for patients with ALL. These data further indicate that the co-stimulatory molecules are involved in multiple aspects of immune activity and susceptibility of CMV infection in transplantation.

Notably, the SNPs of CTLA4 and PDCD1 are associated with several adverse outcomes in patients with AML or ALL. Four SNPs in the CTLA4 gene are associated with the risk for mortality (rs733618, rs11571316, rs3087243, and rs11571315). These SNPs are also known to associate with autoimmune diseases and cancers (39). Another five SNPs in the CTLA4 gene are related to the status of GVHD (rs5742909, rs4553808, rs62182595, rs16840252, and rs231775). Among these SNPs, rs733618, rs11571315, rs11571316, rs5742909, rs4553808, rs62182595, and rs16840252 are within the promoter region, rs231775 is in exon 1, and rs3087243 is in the 3’-UTR of CTLA4 gene. Because CTLA4 expression is important to evade surveillance from host immune cells (40), these SNPs are likely to modulate CTLA4 gene expression, thereby altering the immune response and conferring a risk for mortality post-HSCT. Consistent with this notion, the SNPs located on the promoter region have been shown to elicit effects on gene expression (41), The genotypic variants of rs3087243 have been shown to associate with CTLA4 expression in patients with inflammatory bowel disease (42). The A allele of rs231775 is known to produce higher mRNA efficiency than the G allele, leading to produce more CTLA4 protein (30). It is worthy to investigate further whether the abovementioned SNPs regulate CTLA4 expression leading to aGVHD and cGVHD.

Five SNPs (rs36084323, rs5839828, rs41386349, rs6705653, and rs2227982) in PDCD1 gene are associated with the risk for relapse, mortality, CMV infection, and GVHD. This is consistent with the key roles of PDCD1 in regulating allogeneic immune response in transplantation. Maintenance of graft tolerance is related to the interaction between PDCD1 (PD-1) and PD-L (43). Post-transplantation lymphoproliferative disorder, which was developed under the condition of T-cell dysfunction or immunosuppression after HSCT, is also related to the expression of PDCD1 (44). Among the SNPs, rs5839828 and rs36084323 are within the promoter region, rs6705653 and rs41386349 are in intron 4, and rs2227982 is in exon 5. The SNPs located in the promoter and exon regions may affect the expression of transcription and the alteration of coding amino acid, respectively. Whether intronic SNP has any effect on PDCD1 expression is not clear. Nevertheless, aberrant splicing has been linked to the intronic SNP and causes protein mutation (45).

Four SNPs (rs200353921, rs3181096, rs3181098, and rs28541784) in CD28 gene are associated with the GVHD grades and relapse for patients with AML and ALL. These SNPs may directly or indirectly alter CD28 expression to induce different degrees of cellular responses (46), which, in turn, affect the risk of GVHD and relapse for leukemic patients after HSCT. The interplay between CD28 and GVHD has been reported in several previous studies. CD28 in donor T cells contributes to the pathogenesis and severity of GVHD in a mouse model (17). Abnormal expression of CD28 and CTLA4 in peripheral blood leukocytes of patients with AML may promote the development of aGVHD after HSCT (47). Consistent with these previous studies, our data revealed that the donor SNPs in CD28 gene were related to the development of GVHD in AML patients, regardless of the grade status. Moreover, two SNPs (rs45454293 and rs1234314) in the promoter region of TNFSF4 gene are associated with the development of GVHD grades III and IV for patients with AML. In accord with our findings, Tripathi et al. showed that OX40L (TNFSF4)–OX40 interaction not only induces aGVHD, but also is an essential part in the progression of aGVHD (48). The SNPs in the promoter region is likely to modulate OX40L (TNFSF4) expression, resulting in excessive OX40L–OX40 interaction, which subsequently increases the risk of GVHD.

In addition to genetic studies to associate SNPs with the prognosis of leukemia patients post-HSCT, studies have been reported to integrate both clinical variables and genetic variables in generating predictive model for clinical outcomes after HSCT (49–51). In this regard, Martinez-Laperche et al. applied a complex estimation method, the least absolute shrinkage and selection operation (LASSO) procedure, to generate a predictive model to improve the prediction of severe GVHD (grades III–IV) (49). The model including both clinical variables and genetic variables is better than the models containing only clinical variables or only genetic polymorphisms. Another risk model integrating SNPs and clinical variables have also been demonstrated to predict the risk for GVHD in specific organs (50, 51). An extension of the current study is to integrate clinical variables with our SNP data for multivariate regression analysis and association study. Increasing the enrollment number of donor–recipient pairs may further validate and confirm the importance of these SNPs in the development of adverse outcomes post-HSCT for patients with leukemia.

In conclusion, a total of 10 and 12 SNPs in the co-stimulatory genes are associated with the post-HSCT adverse outcomes for patients with AML and ALL, respectively. Because these SNPs are present in the donor DNA, it provides a basis for developing a screening panel of SNPs to search and select appropriate donors for transplantation. It is also worthy to investigate further the effects of these SNPs on the expression of these co-stimulatory genes to elucidate the underlying mechanisms of transplantation failure.

## Data Availability Statement

The datasets presented in this study can be found in online repositories. The names of the repository/repositories and accession number(s) can be found in the article/**Supplementary Material**.

## Ethics Statement

The studies involving human participants were reviewed and approved by the Institutional Review Board of Chang Gung Memorial Hospital. The patients/participants provided their written informed consent to participate in this study.

## Author Contributions

D-PC conceived and designed the experiments. P-NW provided laboratory samples. D-PC, W-TL, and C-PT wrote the draft of the manuscript. F-PH performed the experiments and analyzed the data. W-TW reviewed and approved the final draft. S-WC and C-PT reviewed literature, and analyzed and interpreted data. All authors contributed to the article and approved the submitted version.

## Funding

This study was supported by grants to D-PC from the Chang Gung Memorial Hospital (CMRPG3H0011, CMRPG3H0012, and CMRPG3H0013) and the Ministry of Science and Technology (109-2320-B-182A-012), and to C-PT from the Chang Gung Memorial Hospital (CMRPD1F0611-3, CMRPD1H0211-3, CMRPD1K0071-2, CORPD1K0031, and BMRP466) and the Ministry of Science and Technology (106-2320-B-182-027-MY3, and 109-2320-B-182-031-MY3). The funders had no role in the study design, data collection and analysis, decision to publish, or preparation of the manuscript.

## Conflict of Interest

The authors declare that the research was conducted in the absence of any commercial or financial relationships that could be construed as a potential conflict of interest.

## Publisher’s Note

All claims expressed in this article are solely those of the authors and do not necessarily represent those of their affiliated organizations, or those of the publisher, the editors and the reviewers. Any product that may be evaluated in this article, or claim that may be made by its manufacturer, is not guaranteed or endorsed by the publisher.
