# Supplementary material for: The Association Between Single-Nucleotide Polymorphisms of Co-Stimulatory Genes Within Non-HLA Region and the Prognosis of Leukemia Patients With Hematopoietic Stem Cell Transplantation
Source: Front Immunol. 2021 Oct 4;12:730507. doi: 10.3389/fimmu.2021.730507 (PMC8520956; doi:10.3389/fimmu.2021.730507)
Supplement: Supplementary file 1 [file Table_1.doc]

**Table S1. The accession numbers for the SNPs that are associated with the adverse outcomes post-HSCT**

| SNPs | NCBI dbSNP database accession number |
| --- | --- |
| rs45454293 | 5314461011 |
| rs1234314 | 5314461012 |
| rs3181096 | 5314461014 |
| rs3181098 | 5314461016 |
| rs28541784 | 5314461019 |
| rs200353921 | 5314461022 |
| rs11571315 | 5314461023 |
| rs733618 | 5314461024 |
| rs4553808 | 5314461025 |
| rs11571316 | 5314461026 |
| rs62182595 | 5314461027 |
| rs16840252 | 5314461029 |
| rs5742909 | 5314461031 |
| rs231775 | 5314461032 |
| rs3087243 | 5314461038 |
| rs2227982 | 5314461040 |
| rs6705653 | 5314461041 |
| rs41386349 | 5314461042 |
| rs36084323 | 5314461044 |
| rs5839828 | 5314461045 |
