# Supplementary material for: The Association Between Single-Nucleotide Polymorphisms of Co-Stimulatory Genes Within Non-HLA Region and the Prognosis of Leukemia Patients With Hematopoietic Stem Cell Transplantation
Source: Front Immunol. 2021 Oct 4;12:730507. doi: 10.3389/fimmu.2021.730507 (PMC8520956; doi:10.3389/fimmu.2021.730507)
Supplement: Supplementary file 2 [file Table_2.doc]

**Table S2**. Genotype and allele frequencies of the CTLA4 gene in 163 donors

| **Polymorphism** | **All donors (%)** | | **Donors for ALL (%)** | | **Donors for AML (%)** | |
| --- | --- | --- | --- | --- | --- | --- |
| No of donors | **163** | | **64** | | **99** | |
| **rs11571315** |  |  |  |  |  |  |
| CC | 20 | (12.3) | 11 | (17.2) | 9 | (9.1) |
| TT | 72 | (44.2) | 26 | (40.6) | 46 | (46.5) |
| CT | 64 | (39.3) | 26 | (40.6) | 38 | (38.4) |
| Unknown | 7 | (4.3) | 1 | (1.6) | 6 | (6.1) |
| C allele | 104 | (31.9) | 48 | (37.5) | 56 | (28.3) |
| T allele | 208 | (63.8) | 78 | (60.9) | 130 | (65.7) |
| Unknown | 14 | (4.3) | 2 | (1.6) | 12 | (6.1) |
| **rs733618** |  |  |  |  |  |  |
| CC | 38 | (23.3) | 12 | (18.8) | 26 | (26.3) |
| TT | 54 | (33.1) | 24 | (37.5) | 30 | (30.3) |
| CT | 65 | (39.9) | 28 | (43.8) | 37 | (37.4) |
| Unknown | 6 | (3.7) | 0 | (0.0) | 6 | (6.1) |
| C allele | 141 | (43.3) | 52 | (40.6) | 89 | (44.9) |
| T allele | 173 | (53.1) | 76 | (59.4) | 97 | (49.0) |
| Unknown | 12 | (3.7) | 0 | (0.0) | 12 | (6.1) |
| **rs4553808** |  |  |  |  |  |  |
| AA | 139 | (85.3) | 52 | (81.3) | 87 | (87.9) |
| GG | 2 | (1.2) | 1 | (1.6) | 1 | (1.0) |
| AG | 19 | (11.7) | 11 | (17.2) | 8 | (8.1) |
| Unknown | 3 | (1.8) | 0 | (0.0) | 3 | (3.0) |
| A allele | 297 | (91.1) | 115 | (89.8) | 182 | (91.9) |
| G allele | 23 | (7.1) | 13 | (10.2) | 10 | (5.1) |
| Unknown | 6 | (1.8) | 0 | (0.0) | 6 | (3.0) |
| **rs11571316** |  |  |  |  |  |  |
| AA | 10 | (6.1) | 5 | (7.8) | 5 | (5.1) |
| GG | 82 | (50.3) | 32 | (50.0) | 50 | (50.5) |
| AG | 67 | (41.1) | 26 | (40.6) | 41 | (41.4) |
| Unknown | 4 | (2.5) | 1 | (1.6) | 3 | (3.0) |
| A allele | 87 | (26.7) | 36 | (28.1) | 51 | (25.8) |
| G allele | 231 | (70.9) | 90 | (70.3) | 141 | (71.2) |
| Unknown | 8 | (2.5) | 2 | (1.6) | 6 | (3.0) |
| **rs62182595** |  |  |  |  |  |  |
| AA | 2 | (1.2) | 1 | (1.6) | 1 | (1.0) |
| GG | 143 | (87.7) | 53 | (82.8) | 90 | (90.9) |
| AG | 14 | (8.6) | 10 | (15.6) | 4 | (4.0) |
| Unknown | 4 | (2.5) | 0 | (0.0) | 4 | (4.0) |
| A allele | 18 | (5.5) | 12 | (9.4) | 6 | (3.0) |
| G allele | 300 | (92.0) | 116 | (90.6) | 184 | (92.9) |
| Unknown | 8 | (2.5) | 0 | (0.0) | 8 | (4.0) |
| **rs16840252** |  |  |  |  |  |  |
| CC | 142 | (87.1) | 52 | (81.3) | 90 | (90.9) |
| TT | 2 | (1.2) | 1 | (1.6) | 1 | (1.0) |
| CT | 15 | 9.(2) | 10 | (15.6) | 5 | (5.1) |
| Unknown | 4 | 2.(5) | 1 | (1.6) | 3 | (3.0) |
| C allele | 299 | (91.7) | 114 | (89.1) | 185 | (93.4) |
| T allele | 19 | 5.(8) | 12 | (9.4) | 7 | (3.5) |
| Unknown | 8 | (2.5) | 2 | (1.6) | 6 | (3.0) |
| **rs5742909** |  |  |  |  |  |  |
| CC | 136 | (83.4) | 51 | (79.7) | 85 | (85.9) |
| TT | 2 | (1.2) | 1 | (1.6) | 1 | (1.0) |
| CT | 20 | (12.3) | 11 | (17.2) | 9 | (9.1) |
| Unknown | 5 | (3.1) | 1 | (1.6) | 4 | (4.0) |
| C allele | 292 | (89.6) | 113 | (88.3) | 179 | (90.4) |
| T allele | 24 | (7.4) | 13 | (10.2) | 11 | (5.6) |
| Unknown | 10 | (3.1) | 2 | (1.6) | 8 | (4.0) |
| **rs231775** |  |  |  |  |  |  |
| AA | 20 | (12.3) | 12 | (18.8) | 8 | (8.1) |
| GG | 66 | (40.5) | 25 | (39.1) | 41 | (41.4) |
| AG | 69 | (42.3) | 24 | (37.5) | 45 | (45.5) |
| Unknown | 8 | (4.9) | 3 | (4.7) | 5 | (5.1) |
| A allele | 109 | (33.4) | 48 | (37.5) | 61 | (30.8) |
| G allele | 201 | (61.7) | 74 | (57.8) | 127 | 964.1) |
| Unknown | 16 | (4.9) | 6 | (4.7) | 10 | (5.1) |
| **rs3087243** |  |  |  |  |  |  |
| AA | 10 | (6.1) | 5 | (7.8) | 5 | (5.1) |
| GG | 85 | (52.1) | 35 | (54.7) | 50 | (50.5) |
| AG | 65 | (39.9) | 24 | (37.5) | 41 | (41.4) |
| Unknown | 3 | (1.8) | 0 | (0.0) | 3 | (3.0) |
| A allele | 85 | (26.1) | 34 | (26.6) | 51 | (25.8) |
| G allele | 235 | (72.1) | 94 | (73.4) | 141 | (71.2) |
| Unknown | 6 | (1.8) | 0 | (0.0) | 6 | (3.0) |
