# Supplementary material for: The Association Between Single-Nucleotide Polymorphisms of Co-Stimulatory Genes Within Non-HLA Region and the Prognosis of Leukemia Patients With Hematopoietic Stem Cell Transplantation
Source: Front Immunol. 2021 Oct 4;12:730507. doi: 10.3389/fimmu.2021.730507 (PMC8520956; doi:10.3389/fimmu.2021.730507)
Supplement: Supplementary file 3 [file Table_3.doc]

**Table S3. Genotype and allele frequencies of the TNFSF4 gene in 163 donors**

| **Polymorphism** | **All donors (%)** | | **Donors for ALL (%)** | | **Donors for AML (%)** | |
| --- | --- | --- | --- | --- | --- | --- |
| No of donors | 163 | | 64 | | 99 | |
| **rs1234314** |  |  |  |  |  |  |
| CC | 33 | (20.2) | 15 | (23.4) | 18 | (18.2) |
| GG | 40 | (24.5) | 10 | (15.6) | 30 | (30.3) |
| CG | 77 | (47.2) | 36 | (56.3) | 41 | (41.4) |
| Unknown | 13 | (8.0) | 3 | (4.7) | 10 | (10.1) |
| C allele | 143 | (43.9) | 66 | (51.6) | 77 | (38.9) |
| G allele | 157 | (48.2) | 56 | (43.8) | 101 | (51.0) |
| Unknown | 26 | (8.0) | 6 | (4.7) | 20 | (10.1) |
| **rs45454293** |  |  |  |  |  |  |
| CC | 97 | (59.5) | 39 | (60.9) | 58 | (58.6) |
| TT | 6 | (3.7) | 2 | (3.1) | 4 | (4.0) |
| CT | 46 | (28.2) | 20 | (31.3) | 26 | (26.3) |
| Unknown | 14 | (8.6) | 3 | (4.7) | 11 | (11.1) |
| C allele | 240 | (73.6) | 98 | (76.6) | 142 | (71.7) |
| T allele | 58 | (17.8) | 24 | (18.8) | 34 | (17.2) |
| Unknown | 28 | (8.6) | 6 | (4.7) | 22 | (11.1) |
