# Supplementary material for: The Association Between Single-Nucleotide Polymorphisms of Co-Stimulatory Genes Within Non-HLA Region and the Prognosis of Leukemia Patients With Hematopoietic Stem Cell Transplantation
Source: Front Immunol. 2021 Oct 4;12:730507. doi: 10.3389/fimmu.2021.730507 (PMC8520956; doi:10.3389/fimmu.2021.730507)
Supplement: Supplementary file 4 [file Table_4.doc]

**Table S4. Genotype and allele frequencies of the CD28 gene in 163 donors**

| **Polymorphism** | **All donors (%)** | | **Donors for ALL (%)** | | **Donors for AML (%)** | |
| --- | --- | --- | --- | --- | --- | --- |
| No of donors | **163** | | **64** | | **99** | |
| **rs3181096** |  |  |  |  |  |  |
| CC | 92 | (56.4) | 38 | (59.4) | 54 | (54.5) |
| TT | 18 | (11.0) | 11 | (17.2) | 7 | (7.1) |
| CT | 42 | (25.8) | 11 | (17.2) | 31 | (31.3) |
| Unknown | 11 | (6.7) | 4 | (6.3) | 7 | (7.1) |
| C allele | 226 | (69.3) | 87 | (68.0) | 139 | (70.2) |
| T allele | 78 | (23.9) | 33 | (25.8) | 45 | (22.7) |
| Unknown | 22 | (6.7) | 8 | (6.3) | 14 | (7.1) |
| **rs3181098** |  |  |  |  |  |  |
| AA | 19 | (11.7) | 12 | (18.8) | 7 | (7.1) |
| GG | 89 | (54.6) | 37 | (57.8) | 52 | (52.5) |
| AG | 40 | (24.5) | 10 | (15.6) | 30 | (30.3) |
| Unknown | 15 | (9.2) | 5 | (7.8) | 10 | (10.1) |
| A allele | 78 | (23.9) | 34 | (26.6) | 44 | (22.2) |
| G allele | 218 | (66.9) | 84 | (65.6) | 134 | (67.7) |
| Unknown | 30 | (9.2) | 10 | (7.8) | 20 | (10.1) |
| **rs28541784** |  |  |  |  |  |  |
| CC | 94 | (57.7) | 36 | (56.3) | 58 | (58.6) |
| TT | 16 | (9.8) | 5 | (7.8) | 11 | (11.1) |
| CT | 35 | (21.5) | 17 | (26.6) | 18 | (18.2) |
| Unknown | 18 | (11.0) | 6 | (9.4) | 12 | (12.1) |
| C allele | 223 | (68.4) | 89 | (69.5) | 134 | (67.7) |
| T allele | 67 | (20.6) | 27 | (21.1) | 40 | (20.2) |
| Unknown | 36 | (11.0) | 12 | (9.4) | 24 | (12.1) |
| **rs200353921** |  |  |  |  |  |  |
| AA | 41 | (25.2) | 18 | (28.1) | 23 | (23.2) |
| TT | 89 | (54.6) | 32 | (50.0) | 57 | (57.6) |
| AT | 14 | (8.6) | 8 | (12.5) | 6 | (6.1) |
| Unknown | 19 | (11.7) | 6 | (9.4) | 13 | (13.1) |
| A allele | 96 | (29.4) | 44 | (34.4) | 52 | (26.3) |
| T allele | 192 | (58.9) | 72 | (56.3) | 120 | (60.6) |
| Unknown | 38 | (11.7) | 12 | (9.4) | 26 | (13.1) |
