# Supplementary material for: The Association Between Single-Nucleotide Polymorphisms of Co-Stimulatory Genes Within Non-HLA Region and the Prognosis of Leukemia Patients With Hematopoietic Stem Cell Transplantation
Source: Front Immunol. 2021 Oct 4;12:730507. doi: 10.3389/fimmu.2021.730507 (PMC8520956; doi:10.3389/fimmu.2021.730507)
Supplement: Supplementary file 5 [file Table_5.doc]

**Table S5.** Genotype and allele frequencies of the PDCD1 gene in 163 donors

| **Polymorphism** | **All donors (%)** | | **Donors in ALL (%)** | | **Donors in AML (%)** | |
| --- | --- | --- | --- | --- | --- | --- |
| No of donors | **163** | | **64** | | **99** | |
| **rs5839828** |  |  |  |  |  |  |
| G7 | 26 | (16.0) | 11 | (17.2) | 15 | (15.2) |
| G6 | 55 | (33.7) | 21 | (32.8) | 34 | (34.3) |
| G6/G7 | 68 | (41.7) | 26 | (40.6) | 42 | (42.4) |
| Unknown | 14 | (8.6) | 6 | (9.4) | 8 | (8.1) |
| G7 allele | 120 | (36.8) | 48 | (37.5) | 72 | (36.4) |
| G6 allele | 178 | (54.6) | 68 | (53.1) | 110 | (55.6) |
| Unknown | 28 | (8.6) | 12 | (9.4) | 16 | (8.1) |
| **rs36084323** |  |  |  |  |  |  |
| CC | 36 | (22.1) | 15 | (23.4) | 21 | (21.2) |
| TT | 42 | (25.8) | 17 | (26.6) | 25 | (25.3) |
| CT | 66 | (40.5) | 24 | (37.5) | 42 | (42.4) |
| Unknown | 19 | (11.7) | 8 | (12.5) | 11 | (11.1) |
| C allele | 138 | (42.3) | 54 | (42.2) | 84 | (42.4) |
| T allele | 150 | (46.0) | 58 | (45.3) | 92 | (46.5) |
| Unknown | 38 | (11.7) | 16 | (12.5) | 22 | (11.1) |
| **rs41386349** |  |  |  |  |  |  |
| AA | 8 | (4.9) | 3 | (4.7) | 5 | (5.1) |
| GG | 92 | (56.4) | 38 | (59.4) | 54 | (54.5) |
| AG | 42 | (25.8) | 15 | (23.4) | 27 | (27.3) |
| Unknown | 21 | (12.9) | 8 | (12.5) | 13 | (13.1) |
| A allele | 58 | (17.8) | 21 | (16.4) | 37 | (18.7) |
| G allele | 226 | (69.3) | 91 | (71.1) | 135 | (68.2) |
| Unknown | 42 | (12.9) | 16 | (12.5) | 26 | (13.1) |
| **rs6705653** |  |  |  |  |  |  |
| CC | 72 | (44.2) | 26 | (40.6) | 46 | (46.5) |
| TT | 16 | (9.8) | 7 | (10.9) | 9 | (9.1) |
| CT | 49 | (30.1) | 20 | (31.3) | 29 | (29.3) |
| Unknown | 26 | (16.0) | 11 | (17.2) | 15 | (15.2) |
| C allele | 193 | (59.2) | 72 | (56.3) | 121 | (61.1) |
| T allele | 81 | (24.8) | 34 | (26.6) | 47 | (23.7) |
| Unknown | 52 | (16.0) | 22 | (17.2) | 30 | (15.2) |
| **rs2227982** |  |  |  |  |  |  |
| AA | 35 | (21.5) | 15 | (23.4) | 20 | (20.2) |
| GG | 40 | (24.5) | 18 | (28.1) | 22 | (22.2) |
| AG | 57 | (35.0) | 17 | (26.6) | 40 | (40.4) |
| Unknown | 31 | (19.0) | 14 | (21.9) | 17 | (17.2) |
| A allele | 127 | (39.0) | 47 | (36.7) | 80 | (40.4) |
| G allele | 137 | (42.0) | 53 | (41.4) | 84 | (42.4) |
| Unknown | 62 | (19.0) | 28 | (21.9) | 34 | (17.2) |
